# Quality of Life and Coping in Nonalcoholic Fatty Liver Disease: Influence of Diabetes and Obesity

**DOI:** 10.3390/ijerph18073503

**Published:** 2021-03-28

**Authors:** Jesús Funuyet-Salas, María Ángeles Pérez-San-Gregorio, Agustín Martín-Rodríguez, Manuel Romero-Gómez

**Affiliations:** 1 Department of Personality, Assessment, and Psychological Treatment, Faculty of Psychology, University of Seville, 41018 Seville, Spain; anperez@us.es (M.Á.P.-S.-G.); amartinr@us.es (A.M.-R.); 2 UCM Digestive Diseases and Ciberehd, Institute of Biomedicine of Seville, Virgen del Rocío University Hospital, University of Seville, 41013 Seville, Spain; mromerogomez@us.es

**Keywords:** NAFLD, quality of life, coping strategies, type 2 diabetes mellitus, obesity

## Abstract

Our aim was to analyze how type 2 diabetes and obesity influence quality of life (QoL) and coping in patients with nonalcoholic fatty liver disease (NAFLD), and which coping strategies predict diabetic or obese participants’ QoL. QoL (SF-12, CLDQ-NAFLD) and coping strategies (COPE-28) were evaluated in 307 biopsy-proven NAFLD patients with absence or presence of diabetes or obesity. QoL was compared with normality tables for the general Spanish population. Interactive effects were found in physical functioning (*p* = 0.008), role-physical (*p* = 0.016) and activity (*p* = 0.014). Diabetic patients reported worse scores when they were also obese and vice versa, that is, obese patients scored worse when they were also diabetic. Both diabetic and obese patients had lower QoL than those without metabolic pathology or the general population, and obese patients also reported more passive/avoidance coping. Active coping, positive reframing and acceptance predicted better QoL, while denial, self-blame, self-distraction, disengagement and religion predicted lower QoL. In conclusion, diabetes and obesity were associated with lower QoL in patients with NAFLD. Obesity was also associated with more passive/avoidance coping. Furthermore, passive/avoidance coping strategies predicted lower QoL than active, recommending modification of maladaptive coping strategies in future multidisciplinary NAFLD treatments.

## 1. Introduction

Nonalcoholic fatty liver disease (NAFLD) is a wide clinical spectrum spanning from hepatic steatosis to nonalcoholic steatohepatitis, and can progress to different degrees of hepatic fibrosis, cirrhosis and hepatocellular carcinoma [1]. In recent years, NAFLD has become an alarming public health problem as the most common worldwide cause of chronic hepatopathy [2]. Its prevalence is exponentially increasing at the same rate as type 2 diabetes mellitus (T2DM) and obesity, as a consequence of a life style based on unhealthy eating habits and sedentarism [3]. In fact, NAFLD, considered as the hepatic manifestation of metabolic syndrome, has a close two-way relationship with T2DM and obesity. On one hand, NAFLD is highly prevalent among diabetics and the obese, and worsens the complications derived from these pathologies [4,5]. On the other, the presence of T2DM and obesity in patients with NAFLD favors the progression of liver damage [6,7].

NAFLD is associated with worse quality of life (QoL) than other alcoholic, viral, self-immune or cholestatic hepatopathies [8], or the general population [9]. The impact is mainly felt in physical functioning, which patients often refer to as fatigue or lack of vitality [10]. Evidence of the influence of T2DM and obesity on QoL is contradictory. While some studies have suggested greater deterioration in QoL, mainly physical, associated with the presence of T2DM or obesity along with NAFLD [10,11], others have found no significant difference in absence or presence of either metabolic pathology [9,12].

T2DM and obesity usually impact negatively on patient psychosocial adjustment, and therefore, the most frequent and appropriate coping strategies for stress associated with the disease should be determined [13]. Studies disagree about whether these metabolic pathologies are associated with more use of active coping strategies [14,15], or passive/avoidance [16,17]. The relevance of this issue stems from active coping usually predicting better global QoL in both diabetics and obese than avoidance coping [15,18]. The same trend is observed in chronic hepatic patients [19], although for the moment there are no results available for NAFLD.

In this context, we analyzed the differences in QoL and coping strategies of patients with NAFLD by assessing whether T2DM and obesity were present or not, employing data from the general Spanish population to compare QoL. We also determined what coping strategies predicted QoL in both diabetics and obese. We hypothesized that patients would have worse QoL and more passive/avoidance coping when they had T2DM or obesity, and passive/avoidance coping strategies would predict worse QoL than active coping in these patients.

## 2. Materials and Methods

### 2.1. Participants

The sample of this cross-sectional study consisted of 307 patients with biopsy-proven NAFLD (192 men and 115 women) with a mean age of 52.6 ± 12.2. Access to patient records was acquired in 2018 to conduct the study. All patients gave their informed consent for participation. This research was approved by the Ethics Committee of the Virgen del Rocío University Hospital of Seville and was conducted in accordance with the 1975 Declaration of Helsinki. Four groups were formed based on the T2DM (G_1_ = absence, G_2_ = presence) and obesity (G_3_ = absence, G_4_ = presence) variables. The sociodemographic characteristics of the groups are shown in Table 1. Data from the general Spanish population (*n* = 4261) for QoL (SF-12) [20] were also considered.

### 2.2. Instruments

*12-Item Short Form Health Survey (SF-12v.2)* [21,22]. This scale evaluates the following health-related QoL dimensions: physical functioning, role-physical, bodily pain, general health, vitality, social functioning, role-emotional and mental health. Using the Quality Metric Health OutcomesTM Scoring Software 5.0 (QualityMetric Incorporated LLC, Johnston, RI, USA), two summary components may be found, the physical component summary (PCS) and mental component summary (MCS). Scores vary from 0 (worst state of health) to 100 (best state of health), where higher scores show better QoL. The Cronbach’s alpha varied from 0.70 to 0.93 for the various dimensions and was 0.92 and 0.88 for the PCS and MCS, respectively [21].

*Chronic Liver Disease Questionnaire-Non-Alcoholic Fatty Liver Disease (CLDQ-NAFLD)* [10]. This measure evaluates the following NAFLD-related QoL dimensions: abdominal symptoms, activity, emotional, fatigue, systemic symptoms and worry, as well as a total score for the scale. Scores vary from 1 to 7, higher scores showing better QoL. The Cronbach’s alpha varied from 0.65 to 0.86 for the different dimensions, and was 0.92 for the total score.

*The Brief COPE (COPE-28)* [23,24]. This evaluates the following coping strategies: active coping, planning, instrumental support, emotional support, self-distraction, venting, disengagement, positive reframing, denial, acceptance, religion, substance use, humor and self-blame. Scores can vary from 0 to 3. Higher scores show more use of the coping strategy. The Cronbach’s alpha varied from 0.74 to 1.00 for the various subscales.

### 2.3. Procedure

**Figure 1:**
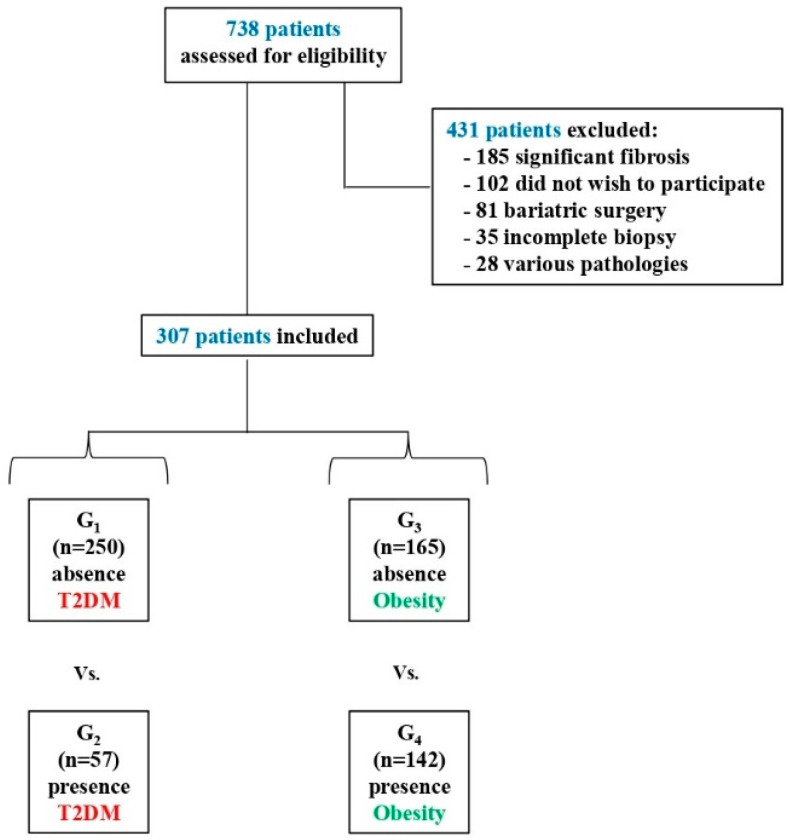
Participant selection for the study.

As shown in Figure 1, 307 patients with NAFLD were selected from 12 Spanish hospitals. Inclusion criteria were: (a) over 18 years old, (b) diagnosis of biopsy-proven NAFLD without significant fibrosis, (c) informed consent, (d) no severe or disabling psychopathological condition and (e) being able to understand the evaluation instruments. Furthermore, keeping in mind the transcendence of significant fibrosis in the biopsychosocial profile of the NAFLD patient, as independently associated with worse QoL and more passive/avoidance coping [25], the presence of significant fibrosis was considered an exclusion criterion in this study. Cancelling out the potential effect of fibrosis on the results enabled a more precise analysis of the influence of the metabolic pathology on the biopsychosocial profile associated with NAFLD. The participants were classified by absence or presence of T2DM and obesity, and evaluated using a psychosocial interview and the SF-12 [21,22], CLDQ-NAFLD [10] and COPE-28 [23,24]. Recruited patients answered the questionnaires prospectively. The questionnaires were filled in by the participants on paper forms, and their data transferred to a common database.

### 2.4. Statistical Analysis

To compare the sociodemographic variables, the Pearson’s Chi-square was applied to the categorical variables (gender, marital status, education and employment), and the independent sample *t*-test for age. A 2 × 2 factorial ANOVA (Snedecor’s *F*) was done to analyze the influence of absence or presence of T2DM and obesity on QoL (SF-12, CLDQ-NAFLD) and coping strategies (COPE-28). Cohen’s *d* (for continuous variables) and *w* (for categorical variables) were computed as effect size indexes. According to Cohen [26], effect sizes can be null (*d*, < 0.2; *w*, < 0.1), small (*d*, ≥ 0.2; *w*, ≥ 0.1), medium (*d*, ≥ 0.5; *w*, ≥ 0.3) or large (*d*, ≥ 0.8; *w*, ≥ 0.5). A stepwise multiple linear regression analysis was performed to analyze what coping strategies predicted QoL (criterion or dependent variable; PCS, MCS, and total CLDQ-NAFLD) in both diabetic and obese patients with NAFLD. A series of statistical parameters were calculated for this: to begin with, the unstandardized (*B*) and standardized (*β*) partial regression coefficients, and their standard error (*SE*). This coefficient reports the relationship between the dependent and the independent variables, so the farther from 0, the stronger the intensity of the relationship. The sign of the coefficient suggests the direction of the relationship: when positive, that the higher the value of the coping strategy is, the higher the quality of life, while if it is negative, it shows that the quality of life decreases with higher value of coping strategy. Furthermore, *t*-test significance was estimated such that a *p* below 0.05 confirmed a statistically significant relationship between the independent variable and the criteria variable. Finally, the coefficient of determination (*R*^2^), which refers to the proportion of variability in the dependent variable explained by the set of independent variables, was found. This coefficient varies from 0 to 1, and the higher it is, the more explanatory the model proposed is. *R*^2^ can be overestimated depending on the number of independent variables in the model, and therefore, it is usually corrected by the number of degrees of freedom, which yields the corrected coefficient of determination (Δ*R*^2^). Statistical requirements for the implementation of linear regression analysis (linearity, independence of residuals, homoscedasticity, and no-multicollinearity) were fulfilled.

## 3. Results

### 3.1. Sociodemographic Variables

In most of the sociodemographic variables (age, gender, marital status, education and employment) there were no important between-groups differences (null or small effect sizes) (Table 1), except diabetic patients (G_2_) were older than nondiabetics (G_1_) (*p* < 0.001, *d* = −0.60, Table 1).

### 3.2. Influence of T2DM and Obesity on QoL and Coping Strategies

The results are shown in Table 2 (SF-12), Table 3 (CLDQ-NAFLD) and Table 4 (COPE-28). Three statistically significant interactive effects were found: physical functioning (*p* = 0.008, Table 2), role-physical (*p* = 0.016, Table 2) and activity (*p* = 0.014, Table 3).

**Figure 2:**
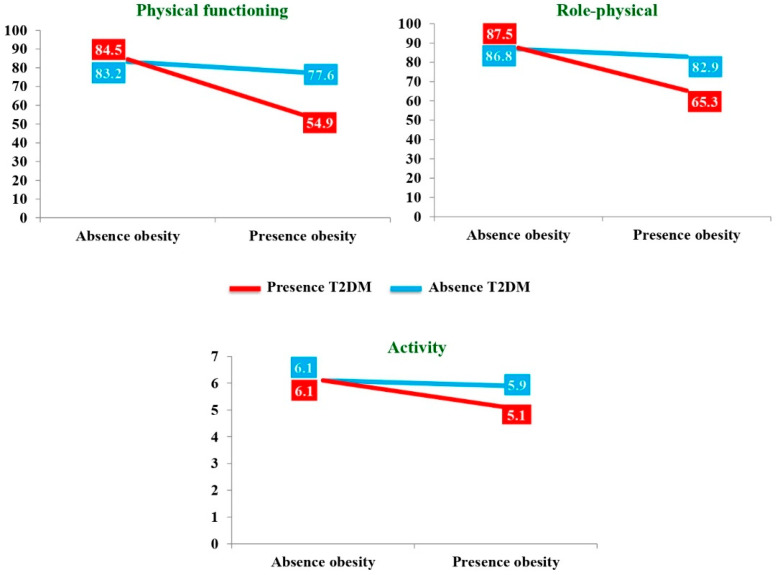
Interactive effects regarding physical functioning, role-physical and activity dimensions in patients with NAFLD.

As observed in Table 4 and in Figure 2, the simple effects showed statistically significant differences, with relevant effect sizes (medium or large), in the groups with T2DM (G_2_) or obesity (G_4_). In particular, diabetic patients showed less physical functioning (*d* = 0.99), role-physical (*d* = 0.89) and activity (*d* = 1.01) if they were obese. Similarly, obese patients had less physical functioning (*d* = 0.76), role-physical (*d* = 0.71) and activity (*d* = 0.79) if they were diabetic.

**Figure 3:**
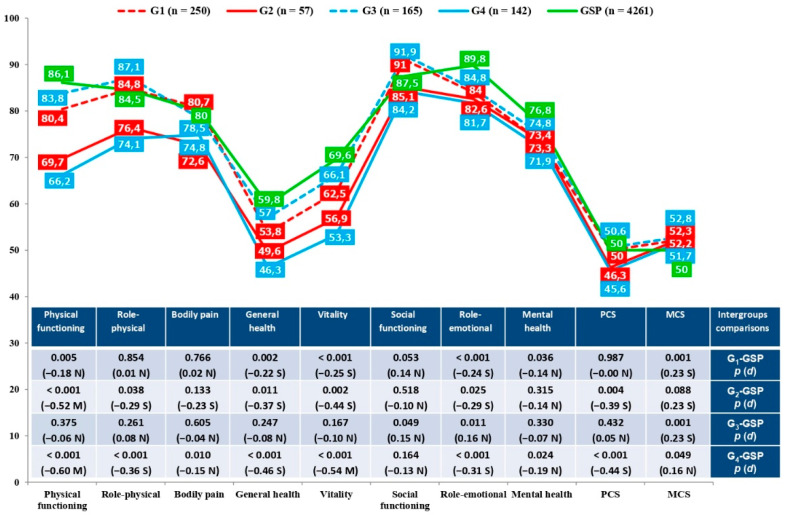
Comparison of quality of life (SF-12) of groups of patients and the general Spanish population. Note. G_1_: absence T2DM; G_2_: presence T2DM; G_3_: absence obesity; G_4_: presence obesity; GSP: general Spanish population; PCS: physical component summary; MCS: mental component summary; N: null effect size; S: small effect size; M: medium effect size.

Concerning the main effects, QoL (SF-12, CLDQ-NAFLD) was worse for diabetic (G_2_) than nondiabetic (G_1_) patients, regardless of absence or presence of obesity. Their scores were lower in physical functioning (*d* = 0.35), role-physical (*d* = 0.33), bodily pain (*d* = 0.31), social functioning (*d* = 0.29), PCS (*d* = 0.40), activity (*d* = 0.39), fatigue (*d* = 0.32), systemic symptoms (*d* = 0.52) and total CLDQ-NAFLD (*d* = 0.35) (Table 2 and Table 3, Figure 3). It was also worse for obese patients (G_4_) than those who were not (G_3_), whether they had T2DM or not, with worse scores in physical functioning (*d* = 0.44), role-physical (*d* = 0.39), general health (*d* = 0.32), vitality (*d* = 0.37), social functioning (*d* = 0.29), PCS (*d* = 0.41), activity (*d* = 0.49), fatigue (*d* = 0.25) and total CLDQ-NAFLD (*d* = 0.32) (Table 2 and Table 3, Figure 3). The main differences from the general Spanish population (GSP) were in the comparison with diabetic (G_2_) and obese (G_4_) patients, who had worse QoL in physical functioning (T2DM, *d* = −0.52; obesity, *d* = −0.60), role-physical (T2DM, *d* = −0.29; obesity, *d* = −0.36), general health (T2DM, *d* = −0.37; obesity, *d* = −0.46), vitality (T2DM, *d* = −0.44; obesity, *d* = −0.54), role-emotional (T2DM, *d* = −0.29; obesity, *d* = −0.31) and PCS (T2DM, *d* = −0.39; obesity, *d* = −0.44) (Figure 3). Nevertheless, no statistically significant differences were found in the main effects of coping strategies (COPE-28) between diabetic patients (G_2_) and nondiabetic (G_1_). However, obese patients (G_4_), whether or not they had T2DM, had lower scores in active coping (*d* = 0.25) and acceptance (*d* = 0.25), and higher in disengagement (*d* = −0.30), than those who were not obese (G_3_) (Table 5).

### 3.3. Coping Strategies Predicting QoL

The results of the multiple linear regression analysis of diabetic (G_2_) and obese (G_4_) patients are presented in Table 6, Table 7 and Table 8. In both groups, the final model (T2DM, *F*_(1, 55)_ = 12.50, *p* = 0.001; obesity, *F*_(1, 140)_ = 16.10, *p* < 0.001) consisted of one significant PCS (SF-12) predictor: in diabetics, denial (*p* = 0.001), and in obese patients active coping (*p* < 0.001). This model explained 18.5% and 10.3% of the variance observed in PCS (SF-12) in diabetic and obese patients, respectively (Table 6). 

Concerning MCS (SF-12), the final model (T2DM, *F*_(4, 52)_ = 8.53, *p* < 0.001; obesity, *F*_(3, 138)_ = 32.53, *p* < 0.001) consisted of four predictors for diabetics (G_2_) and three for obese (G_4_) patients. In diabetics these were acceptance (*p* = 0.009), self-distraction (*p* = 0.034), disengagement (*p* = 0.029) and religion (*p* = 0.030), and in obese patients, positive reframing (*p* < 0.001), self-blame (*p* < 0.001) and denial (*p* = 0.006). This model explained 39.6% and 41.4% of the variance observed in MCS (SF-12) in diabetic and obese patients, respectively (Table 7).

The final CLDQ-NAFLD model (T2DM, *F*_(2, 54)_ = 18.53, *p* < 0.001; obesity, *F*_(3, 138)_ = 22.11, *p* < 0.001) consisted of two predictors for diabetic (G_2_) and three for obese (G_4_) patients. In both groups, denial (T2DM, *p* < 0.001; obesity, *p* < 0.001). Furthermore, positive reframing (*p* = 0.006) in diabetics, and active coping (*p* < 0.001) and self-blame (*p* = 0.003) in obese patients. This model explained 40.7% and 32.5% of the variance observed in total quality of life (CLDQ-NAFLD) in diabetic and obese patients, respectively (Table 8).

## 4. Discussion

This study analyzed the differences in QoL and coping strategies of NAFLD patients with and without T2DM and obesity. It also determined what coping strategies predicted QoL in diabetic and obese patients with NAFLD. There were no important sociodemographic differences between the groups compared, except age, where diabetic participants were older than those without metabolic pathology, as already observed in other studies [27,28].

Significant interaction effects of T2DM and obesity on QoL, but not on coping strategies, were found in physical functioning, role-physical and activity. An additional analysis revealed that of the diabetic patients, those who were obese scored worse on these three QoL dimensions, as the obese patients with T2DM did. Thus, the combination of both metabolic pathologies predicted worse patient QoL [29], particularly physical. This deterioration in physical functioning and activity had already been recently mentioned in other studies on diabetic and obese populations with NAFLD [10,30].

When patients with and without T2DM were compared, whether obese or not, the diabetics referred to worse QoL, again focusing on physical differences (physical functioning, role-physical, bodily pain, social functioning, PCS, activity, fatigue, systemic symptoms, and total CLDQ-NAFLD). Along the same line, obese patients also reported worse QoL, mainly physical (physical functioning, role-physical, general health, vitality, social functioning, PCS, activity, fatigue, and total CLDQ-NAFLD), than those who were not, regardless of absence or presence of T2DM. Our results therefore contradicted the conclusions of Sayiner et al. (2016), and Tapper and Lai (2016) and agreed with Younossi and Henry (2015) and Younossi et al. (2017), as we confirmed the significant effect of T2DM and obesity on the QoL of patients with NAFLD [9,10,11,12]. Comparison with the general Spanish population ratified this conclusion, with diabetics and obese people showing greater decline in their QoL, mainly their physical health (physical functioning, role-physical, general health, vitality, role-emotional and PCS). In line with previous studies in other countries [31,32], diabetics and obese people generally perceived less functional capacity and energy than healthy people, which is closely associated with characteristic problems in these patients such as resistance to insulin or oxidative stress.

Despite previous evidence noting an impact of T2DM on patient coping [14,16], in our study, absence or presence of T2DM made no difference in the coping strategies employed by participants. Nevertheless, obese patients did have significantly lower scores on active coping and acceptance, and higher on disengagement, than those who were not. Thus, as found by Fettich and Chen (2012), obesity was associated with more passive/avoidance coping [17]. Body dissatisfaction could partly explain these results, as it has been linked with less active coping by the obese based on behavioral disengagement as their main coping strategy [33].

We were also able to confirm that coping strategies predict QoL of patients with NAFLD: in diabetics, denial, and in the obese, active coping predicted PCS; acceptance, self-distraction, disengagement and religion predicted MCS in diabetics, while positive reframing, self-blame, and denial did so in the obese. Finally, denial and positive reframing predicted the total CLDQ-NAFLD in diabetics, while denial, active coping and self-blame did in obese. Our results therefore revealed that an active coping style, focused on action (active coping, positive reframing or acceptance), was associated with better QoL in diabetics and obese people, in line with other studies [15,18]. On the contrary, a passive/avoidance coping style (denial, self-blame, self-distraction or disengagement) was related to greater decline, mainly in the mental QoL, of these patients. This type of coping, more focused on emotion, predicts worse mental health and a higher presence of distress and maladaptive health behavior in people with T2DM or obesity, which implies negative consequences to their QoL [15]. Lastly, religion, which may be active or passive/avoidance, predicted worse QoL in the diabetic participants in this study. Religion as a coping strategy has previously been associated with more depressive symptomatology and self-blame in these patients, who interpret the disease as punishment for what they have done in their lives [34].

Summarizing, this study found differences in the QoL of patients with NAFLD by absence or presence of T2DM and obesity, in which diabetics and obese patients had a worse QoL. There were also differences in coping strategies used by patients by absence or presence of obesity, where obese participants used more passive/avoidance coping. Finally, for the first time, we can confirm the importance of coping strategies in NAFLD: active coping, positive reframing and acceptance predicted better QoL, while denial, self-blame, self-distraction, disengagement and religion predicted worse QoL in these patients. The results of this study are clinically relevant, because they suggest the need for multidisciplinary treatments for patients with NAFLD who have not yet developed significant fibrosis, in which intervention in coping strategies should be a major element. The main goal would be to reduce the use of passive/avoidance strategies associated with more helplessness and demotivation in complying with therapeutic recommendations [19], and therefore, with worse therapeutic adherence. Considering that this is certainly low in patients with NAFLD [35], perception of controllability and confidence in managing the disease and its treatment should be promoted, especially in diabetics and obese people. This would lead them to have more faith in active coping strategies. An active coping style, based on acceptance of the disease and on positive reinterpretation of its implications and treatment, would probably involve stronger commitment and active participation of the patient in the NAFLD intervention plan, based mainly on following the physical activity and diet plans. This would lead to greater weight loss, better clinical evolution, and therefore, better patient quality of life [36].

The implementation of cognitive-behavioral intervention strategies has shown positive effects on coping style and QoL of patients with chronic metabolic disorders [37]. Thus, decision-making and problem-solving could be emphasized, first so patients learn to identify the barriers that keep them from losing weight, and later, to plan, analyze and carry out a series of alternatives for solving these problems; cognitive restructuration, which modifies those cognitive biases and unrealistic expectations for losing weight, promoting a more adaptive way of thinking and improving their functional status; and time management, where times during the day that could be used for cooking healthy food or doing physical exercise are planned with the patient [38]. These techniques could promote active coping in diabetics and obese patients diagnosed with NAFLD, which would contribute to keeping the disease from evolving to its most advanced stages. This becomes especially relevant, since in the coming years, cirrhosis secondary to NAFLD is expected to rank as the first cause of liver transplant in the world [39]. Furthermore, the absence of effective pharmacological therapies in the treatment of NAFLD [40] justifies the need to promote a multidisciplinary approach to NAFLD intervention, in which psychological biomarkers would be an important target.

Our study had some limitations. For example, possible collinearity with age in T2DM. Variables such as self-efficacy, responsibility for health or therapeutic adherence could also have affected the relationship of QoL and coping described in this study, and its analysis would be important to future multidisciplinary treatment of NAFLD. Finally, normative QoL data for the general Spanish population were obtained from a cohort from a single Spanish region (20). However, the large size of the sample, made up of patients from real clinical practice in several different Spanish hospitals, constitutes a major strength of this study, and all the participants were biopsy-proven NAFLD patients, which provides added value to the validity of the study results.

## 5. Conclusions

This study found differences in the QoL of patients with NAFLD by absence or presence of T2DM and obesity, in which diabetics and obese patients had a worse QoL. There were also differences in coping strategies used by patients by absence or presence of obesity, where obese participants used more passive/avoidance coping. Finally, for the first time, we can confirm the importance of coping strategies in NAFLD: active coping, positive reframing and acceptance predicted better QoL, while denial, self-blame, self-distraction, disengagement and religion predicted worse QoL in these patients. The results suggest the need for multidisciplinary treatments for patients with NAFLD who have not yet developed significant fibrosis, in which intervention in coping strategies should be a major element.

## Figures and Tables

**Table 1:** Intergroup Comparison of Sociodemographic Variables: Type 2 Diabetes Mellitus (T2DM) (Absence and Presence) and Obesity (Absence and Presence).

	T2DM		Intergroup Comparisons	Effect Sizes
	Absence (G_1_) *n* = 250	Presence (G_2_) *n* = 57		
	***M* (*SD*)**	***M* (*SD*)**	***t* (*p*)**	**Cohen’s *d***
Age	51.3 (12.0)	58.3 (11.5)	*t*_(1, 305)_ = −4.04 (<0.001)	−0.60 M
	**%**	**%**	**χ^2^ (*p*)**	**Cohen’s *w***
Gender			χ^2^_(1)_ = 0.64 (0.422)	0.05 N
Male	63.6	57.9		
Female	36.4	42.1		
Marital status			χ^2^_(1)_ = 0.75 (0.386)	0.05 N
With partner	77.2	82.5		
Without partner	22.8	17.5		
Education			χ^2^_(2)_ = 1.92 (0.383)	0.08 N
Low	37.6	47.4		
Medium	31.6	28.1		
High	30.8	24.6		
Employment			χ^2^_(1)_ = 4.39 (0.036)	0.12 S
Working	60.8	45.6		
Not working	39.2	54.4		
	**Obesity**		**Intergroup Comparisons**	**Effect Sizes**
	**Absence** **(G_3_)** ***n* = 165**	**Presence** **(G_4_)** ***n* = 142**		
	***M* (*SD*)**	***M* (*SD*)**	***t* (*p*)**	**Cohen’s *d***
Age	52.0 (12.4)	53.2 (11.9)	*t*_(1, 305)_ = −0.86 (0.391)	−0.10 N
	**%**	**%**	**χ^2^ (*p*)**	**Cohen’s *w***
Gender			χ^2^_(1)_ = 0.04 (0.848)	0.01 N
Male	63.0	62.0		
Female	37.0	38.0		
Marital status			χ^2^_(1)_ = 0.08 (0.780)	−0.02 N
With partner	78.8	77.5		
Without partner	21.2	22.5		
Education			χ^2^_(2)_ = 2.73 (0.256)	0.09 N
Low	43.0	35.2		
Medium	30.9	31.0		
High	26.1	33.8		
Employment			χ^2^_(1)_ = 2.16 (0.142)	0.08 N
Working	61.8	53.5		
Not working	38.2	46.5		

**Note.** N: null effect size; S: small effect size; M: medium effect size. The independent sample *t*-test (age), and Pearson’s Chi-square (categorical variables) were applied.

**Table 2:** Quality of Life (SF-12) of Patients with Nonalcoholic Fatty Liver Disease (NAFLD) by T2DM (Absence and Presence) and Obesity (Absence and Presence) Variables.

SF-12	T2DM *M* ^a^ (*SD*)		Obesity *M* ^a^ (*SD*)		Main Effects		Interaction Effects
	Absence (G_1_) *n* = 250	Presence (G_2_) *n* = 57	Absence (G_3_) *n* = 165	Presence (G_4_) *n* = 142	T2DM *F*_(1, 303)_ *p* (*d*)	Obesity *F*_(1, 303)_ *p* (*d*)	*F*_(1, 303)_ (*p*)
Physical functioning	80.4 (30.4)	69.7 (31.1)	83.8 (45.0)	66.2 (34.4)	5.53 0.019 (0.35 S)	15.03 <0.001 (0.44 S)	7.03 (0.008)
Role-physical	84.8 (25.1)	76.4 (25.8)	87.1 (37.4)	74.1 (28.6)	5.03 0.026 (0.33 S)	11.97 0.001 (0.39 S)	5.89 (0.016)
Bodily pain	80.7 (25.8)	72.6 (26.3)	78.5 (38.1)	74.8 (29.2)	4.46 0.036 (0.31 S)	0.90 0.343 (0.11 N)	0.10 (0.756)
General health	53.8 (24.8)	49.6 (25.4)	57.0 (36.9)	46.3 (28.2)	1.28 0.259 (0.17 N)	8.26 0.004 (0.32 S)	1.15 (0.284)
Vitality	62.5 (25.9)	56.9 (26.5)	66.1 (38.4)	53.3 (29.4)	2.08 0.150 (0.21 S)	10.95 0.001 (0.37 S)	2.93 (0.088)
Social functioning	91.0 (19.9)	85.1 (20.4)	91.9 (29.5)	84.2 (22.6)	3.99 0.047 (0.29 S)	6.79 0.010 (0.29 S)	3.33 (0.069)
Role-emotional	84.0 (24.2)	82.6 (24.8)	84.8 (36.0)	81.7 (27.5)	0.16 0.693 (0.06 N)	0.72 0.398 (0.10 N)	1.47 (0.225)
Mental health	73.3 (22.1)	73.4 (22.6)	74.8 (32.7)	71.9 (25.1)	0.00 0.987 (−0.00 N)	0.78 0.379 (0.10 N)	3.62 (0.058)
PCS	50.0 (9.2)	46.3 (9.4)	50.6 (13.6)	45.6 (10.4)	7.32 0.007 (0.40 S)	13.05 <0.001 (0.41 S)	2.80 (0.095)
MCS	52.24 (9.5)	52.3 (9.7)	52.8 (14.1)	51.7 (10.8)	0.01 0.941 (−0.01 N)	0.62 0.433 (0.09 N)	2.00 (0.159)

**Note.** N: null effect size; S: small effect size; PCS: Physical component summary; MCS: Mental component summary. 2 × 2 factorial ANOVA was applied. ^a^ Higher scores show more quality of life.

**Table 3:** Quality of Life (CLDQ-NAFLD) of Patients with NAFLD by T2DM (Absence and Presence) and Obesity (Absence and Presence) Variables.

CLDQ-NAFLD	T2DM *M* ^a^ (*SD*)		Obesity *M* ^a^ (*SD*)		Main Effects		Interaction Effects
	Absence (G_1_) *n* = 250	Presence (G_2_) *n* = 57	Absence (G_3_) *n* = 165	Presence (G_4_) *n* = 142	T2DM *F*_(1, 303)_ *p* (*d*)	Obesity *F*_(1, 303)_ *p* (*d*)	*F*_(1, 303)_ (*p*)
Abdominal symptoms	5.8 (1.4)	5.7 (1.4)	5.9 (2.0)	5.6 (1.5)	0.27 0.601 (0.08 N)	2.78 0.096 (0.19 N)	0.02 (0.880)
Activity	6.0 (1.1)	5.6 (1.1)	6.1 (1.5)	5.5 (1.2)	7.10 0.008 (0.39 S)	17.76 <0.001 (0.49 S)	6.07 (0.014)
Emotional	5.9 (0.9)	5.8 (1.1)	5.9 (1.5)	5.9 (1.2)	0.63 0.428 (0.12 N)	0.07 0.795 (0.03 N)	0.07 (0.797)
Fatigue	5.7 (1.1)	5.3 (1.2)	5.7 (1.7)	5.3 (1.3)	4.50 0.035 (0.32 S)	4.85 0.028 (0.25 S)	1.81 (0.179)
Systemic symptoms	6.1 (0.8)	5.7 (0.8)	6.0 (1.3)	5.8 (0.9)	11.37 0.001 (0.52 M)	3.72 0.055 (0.21 S)	0.14 (0.710)
Worry	6.4 (0.8)	6.2 (0.7)	6.4 (1.2)	6.3 (0.8)	2.86 0.092 (0.25 S)	0.98 0.323 (0.11 N)	0.01 (0.925)
Total	6.0 (0.8)	5.7 (0.7)	6.0 (1.0)	5.7 (0.8)	6.00 0.015 (0.35 S)	7.17 0.008 (0.32 S)	1.07 (0.303)

**Note.** N: null effect size; S: small effect size; M: medium effect size. 2 × 2 factorial ANOVA was applied. ^a^ Higher scores show more quality of life.

**Table 4:** Simple Effects in Physical Functioning (SF-12), Role-Physical (SF-12) and Activity (CLDQ-NAFLD).

Obesity	Absence T2DM (G_1_) *n* = 250		Presence T2DM (G_2_) *n* = 57	
	*p*	Cohen’s *d*	*p*	Cohen’s *d*
	**Physical Functioning (SF-12)**			
Absence-presence	0.148	0.19 N	<0.001	0.99 L
	**Role-Physical (SF-12)**			
Absence-presence	0.222	0.16 N	0.001	0.89 L
	**Activity (CLDQ-NAFLD)**			
Absence-presence	0.039	0.26 S	<0.001	1.01 L
**T2DM**	**Absence Obesity** **(G_3_)** ***n* = 165**		**Presence Obesity** **(G_4_)** ***n* = 142**	
	***p***	**Cohen’s *d***	***p***	**Cohen’s *d***
	**Physical Functioning (SF-12)**			
Absence-presence	0.846	−0.04 N	<0.001	0.76 M
	**Role-Physical (SF-12)**			
Absence-presence	0.905	−0.03 N	<0.001	0.71 M
	**Activity (CLDQ-NAFLD)**			
Absence-presence	0.897	0.04 N	<0.001	0.79 M

**Note**. N: null effect size; S: small effect size; M: medium effect size; L: large effect size.

**Table 5:** Coping Strategies (COPE-28) of Patients with NAFLD by T2DM (Absence and Presence) and Obesity (Absence and Presence) Variables.

COPE-28	T2DM *M* ^a^ (*SD*)		Obesity *M* ^a^ (*SD*)		Main Effects		Interaction Effects
	Absence (G_1_) *n* = 250	Presence (G_2_) *n* = 57	Absence (G_3_) *n* = 165	Presence (G_4_) *n* = 142	T2DM *F*_(1, 303)_ *p* (*d*)	Obesity *F*_(1, 303)_ *p* (*d*)	*F*_(1, 303)_ (*p*)
Active coping	2.0 (0.8)	2.1 (0.7)	2.2 (1.0)	1.9 (0.8)	0.47 0.495 (−0.09 N)	4.15 0.042 (0.25 S)	1.72 (0.191)
Planning	1.4 (1.1)	1.4 (1.1)	1.5 (1.5)	1.3 (1.2)	0.02 0.898 (0.02 N)	1.62 0.205 (0.15 N)	0.47 (0.494)
Instrumental support	1.2 (0.9)	1.4 (1.1)	1.4 (1.5)	1.2 (1.2)	2.52 0.114 (−0.24 S)	1.67 0.197 (0.14 N)	0.01 (0.902)
Emotional support	1.1 (1.1)	1.2 (1.1)	1.3 (1.5)	1.1 (1.2)	0.96 0.327 (−0.14 N)	1.35 0.247 (0.13 N)	0.00 (1.000)
Self-distraction	0.8 (0.9)	0.8 (1.0)	0.8 (1.4)	0.8 (1.1)	0.22 0.641 (−0.07 N)	0.14 0.707 (0.04 N)	1.30 (0.255)
Venting	1.0 (0.9)	1.1 (1.1)	0.9 (1.5)	1.1 (1.2)	0.29 0.592 (−0.09 N)	1.56 0.213 (−0.14 N)	0.53 (0.468)
Disengagement	0.2 (0.5)	0.2 (0.4)	0.1 (0.6)	0.3 (0.5)	1.20 0.273 (0.15 N)	5.65 0.018 (−0.30 S)	2.47 (0.117)
Positive reframing	1.4 (0.9)	1.5 (1.1)	1.6 (1.5)	1.3 (1.2)	0.50 0.481 (−0.10 N)	3.34 0.069 (0.20 S)	2.77 (0.097)
Denial	0.2 (0.3)	0.2 (0.4)	0.1 (0.5)	0.2 (0.4)	0.00 0.996 (0.00 N)	2.07 0.151 (−0.18 N)	0.73 (0.392)
Acceptance	2.2 (0.6)	2.1 (0.7)	2.2 (1.0)	2.0 (0.8)	0.56 0.455 (0.11 N)	4.55 0.034 (0.25 S)	0.75 (0.388)
Religion	0.9 (1.3)	0.9 (1.2)	0.9 (1.8)	0.9 (1.3)	0.15 0.697 (−0.06 N)	0.01 0.940 (−0.01 N)	0.11 (0.741)
Humor	1.1 (1.1)	1.4 (1.1)	1.3 (1.5)	1.2 (1.2)	2.08 0.150 (−0.21 S)	0.12 0.724 (0.04 N)	0.65 (0.421)
Self-blame	0.5 (0.6)	0.4 (0.7)	0.4 (1.0)	0.5 (0.8)	0.33 0.564 (0.09 N)	0.81 0.368 (−0.11 N)	2.95 (0.087)
Use Abuse	0	0	0	0	0.16 0.689 (0.00 N)	0.16 0.689 (0.00 N)	0.16 (0.689)

**Note.** N: null effect size; S: small effect size. 2 × 2 factorial ANOVA was applied. ^a^ Higher scores show more use of the coping strategy.

**Table 6:** Coping Strategies as Predictors of Physical Component Summary (SF-12).

Predictor Variables	*B*	*SE*	*β*	*t* (*p*)	*R* ^2^	Δ*R*^2^
**T2DM**						
Step 1					0.18	0.17
Denial	−12.26	3.47	−0.43	−3.53 (0.001)		
**Obesity**						
Step 1					0.10	0.10
Active coping	4.18	1.04	0.32	4.01 (<0.001)		

**Note**. A stepwise multiple linear regression analysis was applied.

**Table 7:** Coping Strategies as Predictors of Mental Component Summary (SF-12).

Predictor Variables	*B*	*SE*	*β*	*t* (*p*)	*R* ^2^	Δ*R*^2^
**T2DM**						
Step 4					0.40	0.35
Acceptance	4.11	1.51	0.33	2.73 (0.009)		
Self-distraction	−2.15	0.99	−0.24	−2.17 (0.034)		
Disengagement	−6.47	2.89	−0.27	−2.24 (0.029)		
Religion	−1.80	0.80	−0.24	−2.24 (0.030)		
**Obesity**						
Step 3					0.41	0.40
Positive reframing	3.55	0.66	0.36	5.34 (<0.001)		
Self-blame	−4.36	0.97	−0.32	−4.51(<0.001)		
Denial	−4.83	1.73	−0.20	−2.79 (0.006)		

**Note**. A stepwise multiple linear regression analysis was applied.

**Table 8:** Coping Strategies as Predictors of Quality of Life (Total CLDQ-NAFLD).

Predictor Variables	*B*	*SE*	*β*	*t* (*p*)	*R* ^2^	Δ*R*^2^
**T2DM**						
Step 2					0.41	0.38
Denial	−1.07	0.24	−0.48	−4.44 (<0.001)		
Positive reframing	0.27	0.09	0.31	2.86 (0.006)		
**Obesity**						
Step 3					0.32	0.31
Denial	−0.58	0.16	−0.28	−3.59 (<0.001)		
Active coping	0.30	0.08	0.28	3.78 (<0.001)		
Self-blame	−0.27	0.09	−0.23	−3.05 (0.003)		

**Note**. A stepwise multiple linear regression analysis was applied.

## Data Availability

Data Availability Statements can be found at https://www.mdpi.com/ethics. The raw data supporting the findings of this study will be made available by the corresponding author upon reasonable request.
